# Attention-Augmented LSTM Feed-Forward Compensation for Lever-Arm-Induced Velocity Errors in Transfer Alignment

**DOI:** 10.3390/biomimetics11010032

**Published:** 2026-01-03

**Authors:** Shuang Pan, Guangyao Yan, Dongping Sun, Binghong Liang, Linping Feng

**Affiliations:** Naval Submarine Academy, Qingdao 266000, China; n7k9pp@126.com (S.P.); 15845225980@163.com (G.Y.); bglibianji@126.com (D.S.); cielphantom0604@163.com (B.L.)

**Keywords:** underwater bio-inspired robot, inertial navigation system, transfer alignment, lever-arm effect, Attention-LSTM, AKF

## Abstract

In a mother–child underwater bio-inspired robotic system, the equivalent lever arm between the master and slave inertial navigation systems (INSs) varies with launcher attitude changes and structural flexure. This time-varying lever arm introduces hard-to-model systematic velocity errors that degrade the accuracy and filter convergence of velocity difference-based transfer alignment. Traditional rigid body compensation relies on precise, constant lever-arm parameters and fails when booms, launch tubes, or flexible manipulators undergo appreciable deformation or reconfiguration. To address this, we augment a “velocity–attitude joint matching and innovation-based adaptive Kalman filter (AKF)” framework with an attention-based Long Short-Term Memory (LSTM) feed-forward module. Using only a short, real-time Inertial Measurement Unit (IMU) sequence from the slave INS, the module predicts and compensates the velocity bias induced by the lever arm. Numerical simulations of an underwater bio-inspired robot deployment scenario show that, under typical maneuvers (acceleration, turning, fin-flapping, and S-curve), the proposed method reduces the root-mean-square (RMS) misalignment angle error from about 14.5′ to 5.2′ and the RMS installation error angle from 8.8′ to 3.0′—average reductions of about 64% and 66%, respectively—substantially improving the robustness and practical applicability of transfer alignment under time-varying lever arms and flexible disturbances.

## 1. Introduction

In recent years, underwater bio-inspired robots such as robotic fish, undulating fin vehicles, and multi-modal hybrid propulsion platforms have emerged in rapid succession, demonstrating exceptional maneuverability and environmental adaptability in missions like resource exploration, environmental monitoring, and autonomous inspection of complex zones [1]. Starting from survey studies on undulating propulsion underwater bio-inspired robots, through novel prototypes that combine undulating fins with flapping foils and their modeling validation, to intelligent safe exploration frameworks designed for cluttered reef environments [2], the accumulated work shows that these platforms possess pronounced advantages in low-speed agility, attitude stability, and the ability to traverse convoluted terrain. Within a mother–child underwater bio-inspired robot system, transfer alignment is the key technology that guarantees the vehicle can obtain high-precision attitude and velocity shortly after entering the water. The typical mission architecture places a high-accuracy master INS on the mother platform, while the recipient is a volume-constrained, lower-grade slave INS embedded in the underwater bio-inspired robot [3]. Before launch, the robot is connected to the mother platform through a hybrid rigid–flexible link such as a boom, launch tube, or flexible manipulator. Once the master INS is initialized, it must propagate attitude and velocity states to the slave INS so that rapid alignment is completed before water entry, thereby avoiding the problems of prolonged alignment time and excessive attitude error that would arise underwater where absolute measurements like Global Navigation Satellite System (GNSS) are unavailable.

However, due to significant spatial installation offsets and structural flexibility between the master and slave INSs, the acceleration and angular rate signals measured by the two units differ markedly whenever the mother platform maneuvers or the launch mechanism moves. Consequently, the velocity solution of the slave INS is superimposed with an additional linear velocity component induced by the angular motion of the mother platform—this phenomenon is known as the lever-arm effect [4]. If this error is not properly compensated, it directly corrupts the filter’s estimate of the slave INS attitude error, leading to slow convergence or even filter divergence [5], and ultimately degrading the mission capability of the underwater bio-inspired robot in complex marine environments.

To mitigate the aforementioned velocity errors, various compensation strategies for lever-arm errors have been proposed in engineering practice. Among them, low-pass filtering attempts to remove the vibration errors caused by the lever arm by filtering out high-frequency components. However, it cannot completely eliminate the errors and may also lose valuable dynamic information, thus having limited practical application [6]. Rigid body compensation methods use known lever-arm geometric parameters to calculate the additional linear velocity induced by the body’s angular velocity and then perform compensation. These methods are simple and effective when the lever-arm parameters are precisely known, but on highly flexible platforms like underwater bio-inspired robots, the lever-arm parameters cannot be kept accurately for long [7]. Extended state introduces states related to the lever arm into the filter state vector, treating the lever-arm error as a state to be estimated online. It can address the unknown lever-arm problem to some extent. However, its performance is highly dependent on the body, which needs to apply specific maneuvering excitations to activate the observability of the lever-arm states. When the maneuvering excitation is insufficient, the convergence speed and accuracy of the extended state method are still not satisfactory [8,9]. In addition, some studies have introduced additional sensors to measure the deformation of the lever arm in order to improve the compensation effect. For example, He et al. employed FBG sensors to monitor flexible lever-arm deformation of the wing immediately, compensating the error at the hardware level [10]. However, such solutions increase the system’s volume, weight, and cost, making them difficult to implement for micro-sized underwater bio-inspired robots.

All the above approaches share a tacit premise: within the time horizon of alignment, the lever-arm parameters are treated as either known or slowly estimable. However, for underwater bio-inspired robots, the tail fin, bio-inspired torso, and components such as cables and booms that connect to the mother platform form a structure that couples both rigid and flexible parts. Under the influence of hydrodynamic and thermal loads, these components undergo complex bending and slack behaviors. This results in composite time-varying characteristics of the equivalent lever arm, including unknown initial values, piecewise constants, sudden jumps, and slow drifts. Consequently, compensation models based on simple rigid body assumptions struggle to accurately describe the true error characteristics.

Meanwhile, conventional error modeling that relies on analytical physical models shows clear limitations when structural uncertainties such as sensor errors and calibration biases are high. This has led researchers to explore more data-driven and adaptable strategies. In recent years deep learning, especially the Long Short-Term Memory (LSTM) architecture, has been widely adopted for time-series modeling, offering new ideas for navigation error compensation and for modeling the dynamics of underwater bio-inspired robots. Thanks to its strong nonlinear fitting capability and built-in memory, LSTM can automatically learn complex time-varying mappings from historical Inertial Measurement Units (IMUs) or actuator data, giving it a clear advantage during GNSS outages, under uncertain structural parameters, and in highly nonlinear flow fields [11]. Within the underwater bio-inspired field, surveys on undulating propulsion vehicles have been published [12], deep learning has been used to build time-series surrogate models for the thrust and power of flexible bio-inspired fins [13], and adaptive navigation-and-control frameworks for bio-inspired unmanned underwater vehicles operating in complex environments have been proposed [14]. Work on GNSS/INS integration has likewise demonstrated, from the viewpoints of sensor error compensation and robust fusion, that LSTM and related networks are both effective and robust for inertial error modeling [15,16,17]. Inspired by these results, this paper introduces an LSTM to compensate for the master/slave lever-arm error, allowing the compensation model to learn and predict the systematic velocity error online under diverse underwater maneuvers without requiring precise lever-arm parameters.

In addition to modeling and compensating for the lever-arm errors between the master and slave INS, accurately fusing multi-source observation information on the basis of compensation to achieve high-precision attitude estimation is a key issue in transfer alignment systems for underwater bio-inspired robots. Velocity matching, which constructs observations by comparing the velocity output differences of the master and slave INS in the navigation frame and can effectively reflect attitude misalignment angles, is the most commonly used method for observation construction currently [18]. However, velocity matching is essentially an indirect observation method, and its observation accuracy is limited by the velocity error propagation chain. In particular, when dynamic excitation is insufficient or compensation errors are large, the estimation performance tends to deteriorate easily [19]. In contrast, attitude matching constructs the observation items directly based on the attitude outputs of the two INSs, which has the advantages of a simple configuration and no need for integration [20]. However, when the slave INS has not yet completed initialization and its accuracy is still low, the attitude matching observations are heavily contaminated by noise [21,22]. Based on the above analysis, a velocity–attitude joint matching strategy has been proposed to integrate the advantages of both methods. This approach combines two types of observation information: velocity differences and attitude differences to construct a joint observation model with greater information redundancy and error compensation capability, which can significantly enhance the observability and convergence speed of the filter [23].

Within the joint matching framework, the Kalman filter and its adaptive variants remain the most widely used state estimation tools [24,25]. However, existing adaptive filtering methods generally assume that observation residuals follow a zero-mean Gaussian process, and they lack the ability to model and feed-forward compensate for non-Gaussian disturbances or structural errors. When the system exhibits complex nonlinearities or unmodeled structural errors, such as bending and slack in the rigid–flexible coupling structure of underwater bio-inspired robots, relying solely on observation feedback to adjust covariances can introduce lag, thereby degrading both convergence speed and estimation accuracy.

In summary, this paper proposes a hardware-free, lever-arm-parameter-free fusion framework that combines an attention-boosted LSTM feed-forward compensator with an innovation-adaptive Kalman filter. Taking only the slave IMU stream as input, the attention mechanism dynamically highlights history-critical features, implicitly captures both gradual and sudden lever-arm variations induced by the flexible mother–child linkage, and outputs real-time velocity corrections. These corrections are then fused with velocity–attitude measurements in an adaptive Kalman filter, achieving fast convergence and eliminating the lag inherent in traditional feedback schemes when structural jumps occur. The approach is tailor-made for pre-launch transfer alignment of underwater bio-inspired vehicles, improving adaptability to configuration changes and environmental disturbances without any extra hardware.

The main contributions of this paper are as follows: (1) For the actual launch conditions of mother–child underwater bio-inspired robot systems, we establish an equivalent lever-arm error model that accounts for both launcher attitude changes and structural flexure. On this basis, we propose unified performance metrics, including root-mean-square errors of three-axis misalignment and installation-error angles, for transfer alignment, enabling quantitative comparison of different compensation strategies under time-varying lever arms. (2) To counteract the time-varying velocity errors caused by flexible and unknown lever arms, we present an attention-augmented LSTM feed-forward compensator that predicts and cancels the lever-arm-induced component in the master/slave velocity difference using only a short slave-IMU sequence, combining physics insight with data-driven modeling. (3) Within a velocity–attitude joint-matching and innovation-adaptive Kalman-filter framework, the feed-forward module is tightly coupled with extended-state modeling and adaptive measurement noise tuning. Simulation results show that, compared with conventional physical lever-arm compensation, the proposed method markedly reduces misalignment and installation estimation errors under typical maneuvers and offers superior filter robustness and engineering practicality when lever-arm parameters jump or flexible disturbances intensify.

## 2. Materials and Methods

To provide a theoretical basis for the subsequent compensation algorithm, a model that relates the velocities of the master and slave inertial navigation systems must first be established. Given that in practice the lever arm exhibits both rigid and flexible characteristics, this paper starts from the rigid lever-arm model to derive the velocity relationship between the master and slave INS. Subsequently, accounting for the dynamic deformation of the lever arm caused by structural flexibility, a more realistic flexible lever-arm system model is constructed.

### 2.1. Rigid Lever Arm Static Modeling

In an inertial navigation system, the master INS is typically installed near the center of mass of the body, while the slave INS is installed at a position with a fixed offset relative to the master INS. Due to the presence of a fixed “lever arm” structure between them, when the body undergoes angular motion, an additional linear velocity component is introduced at the location of the slave INS. This additional velocity caused by angular motion is the source of the lever-arm effect [26]. The coordinate systems and notation used in this paper are listed in Table 1.

Based on the kinematics of a rigid body, the position and velocity relationship between the master and slave INS can be derived.

Firstly, the positional relationship between the master and slave INS in the inertial frame can be expressed as

(1) $$r_{s}^{i}=r_{m}^{i}+C_{b}^{i}r_{sm}^{b}$$

By differentiating the above position relationship with respect to time, the velocity relationship between the master and slave INS is obtained:

(2) $$v_{s}^{i}=\frac{dr_{s}^{i}}{dt}=v_{m}^{i}+\frac{d}{dt}\left(C_{b}^{i}r_{sm}^{b}\right)$$

Since $r_{sm}^{b}$ is constant in the body frame, its time derivative is caused by the changes of the body’s attitude. According to the rigid body rotational kinematics formula, we have

(3) $$\frac{d}{dt}\left(C_{b}^{i}r_{sm}^{b}\right)=C_{b}^{i}\left(\omega_{ib}^{b}\times r_{sm}^{b}\right)$$

This can be rearranged to obtain

(4) $$v_{s}^{i}=v_{m}^{i}+C_{b}^{i}\left(\omega_{ib}^{b}\times r_{sm}^{b}\right)$$

When mapped into the navigation frame, the resulting velocity of the slave INS is

(5) $$v_{s}^{n}=v_{m}^{n}+C_{b}^{n}\left(\omega_{ib}^{b}\times r_{sm}^{b}\right)$$

To more completely characterize the velocity error between the master and slave INS caused by the lever arm, the derivative term is further derived based on Equation (5), resulting in the acceleration of the slave INS in the inertial frame:

(6) $$a_{s}^{n}=a_{m}^{n}+C_{b}^{n}\times r_{sm}^{b}\left({\dot{\omega }}_{ib}^{b}+{\left(\omega_{ib}^{b}\right)}^{2}\right)$$

### 2.2. Dynamic Modeling of Flexible Lever Arm

Based on the rigid model, to more accurately characterize the slight positional offsets between the master and slave INS caused by structural flexibility, it is necessary to introduce a perturbation term that reflects structural dynamic changes in the modeling. This perturbation is not arbitrary but is influenced by the physical properties of the structure (such as elasticity, damping, and driving excitation) and historical dynamic factors, exhibiting dynamic behavior with distinct boundaries, continuity, and randomness. To reasonably describe this dynamic stochastic process, some existing literature has proposed using a second-order Markov model for modeling. Lara-Molina et al. noted in their modeling of flexible robotic arms that a second-order Markov model can be employed to capture the dynamic characteristics of robotic arms [27], and Liu et al. confirmed the same point in the attitude control modeling of flexible spacecraft: adopting a second-order Markov model markedly improves both the accuracy and robustness of state estimation [28].

Therefore, in this paper, a perturbation term $\delta r^{b}(t)$ is introduced in the modeling of the flexible lever arm to describe the slight offsets between the master and slave INS caused by structural deformation. Its dynamic evolution is modeled using a second-order Markov process driven by white noise to better conform to the actual dynamic behavior of the lever arm in engineering applications. The expression is as follows:

(7) $$r_{sm}^{b}(t)=r_{sm}^{b}+\delta r^{b}(t)$$

To ensure that the model is both physically reasonable and convenient for integration with the state modeling of the filter, this paper employs a second-order Markov process driven by white noise to model the evolution of the perturbation term, thereby achieving a systematic characterization of the effects of flexible deformation.

Specifically, the perturbation vector $\delta r^{b}(t)$ can be approximately regarded as the cross product of the initial static vector of the lever arm $r_{sm}^{b}$ and a small dynamic deformation angle $\theta_{v}(t)$:

(8) $$\delta r^{b}(t)\approx \theta_{v}(t)\times r_{sm}^{b}$$

Here, $\theta_{v}(t)$ represents a small-angle perturbation vector due to flexible deformation. This paper simplifies the assumption that the perturbation angle undergoes a small deflection around a fixed axis, representing the dominant flexible deformation component caused by the structural primary mode. Its dynamics satisfy the following second-order Markov stochastic process [29]:

(9) $${\ddot{\theta }}_{v}+2\xi \omega_{n}{\dot{\theta }}_{v}+\omega_{n}^{2}\theta_{v}=w(t)$$

Here, $\omega_{n}$ represents the natural frequency of the system, ξ is the damping ratio, and w(t) denotes the white noise excitation. The above equation can be further transformed into the state-space form more commonly used in filter design:

(10) $$\left\{\begin{array}{l} {\dot{\theta }}_{v}=\omega_{v}\\ {\dot{\omega }}_{v}=-2\xi \omega_{n}\omega_{v}-\omega_{n}^{2}\theta_{v}+w(t) \end{array}\right.$$

At this time, due to the presence of the dynamic perturbation of the lever arm, the velocity relationship between the master and slave INS in the navigation frame can be expressed as

(11) $$v_{s}^{n}(t)=v_{m}^{n}(t)+C_{b}^{n}(t)\left[\omega_{ib}^{b}(t)\times \left(r_{sm}^{b}+\delta r^{b}(t)\right)\right]$$

As can be seen from the above equation, the velocity error caused by the flexible lever-arm perturbation is related to the amplitude and dynamic characteristics of the perturbation angle. When the natural frequency is low or the excitation is severe, this error will be significant; otherwise, it can be neglected.

Compared with the velocity expression in Equation (5) of the rigid lever-arm model, which ignores the lever-arm perturbation, Equation (11) provides a more accurate characterization of the velocity error contribution of the flexible connection structure under dynamic excitation by introducing the perturbation term $\theta_{v}(t)$.

Based on the INS architecture described above, this section establishes a lever-arm-induced velocity error model between the master and slave INS. The rigid lever-arm model is first derived to provide an analytical expression of the velocity error. Subsequently, considering the dynamic perturbation introduced by the flexible connection, the deformation behavior is characterized using a Markov process to construct a more realistic lever-arm error model. This provides a theoretical basis for the subsequent introduction of the Attention-LSTM neural network data fitting model and the filter.

## 3. Algorithm Design

Building on the lever-arm error model described above, this paper will develop a data-driven compensation algorithm for lever-arm-induced velocity errors and an adaptive Kalman filter to improve alignment accuracy.

**Figure 1:**
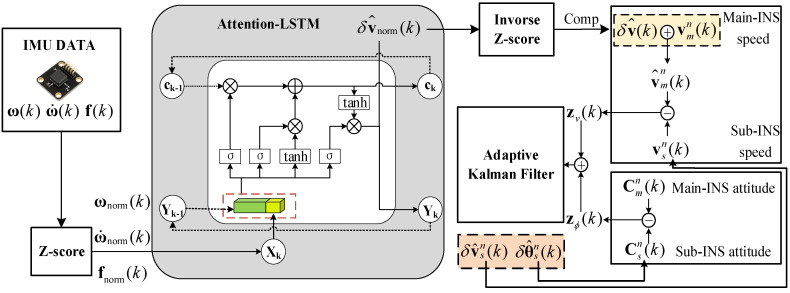
Schematic diagram of transfer alignment using velocity–attitude joint matching with Attention-LSTM prediction.

As illustrated in Figure 1, the framework consists of two main components: an Attention-LSTM compensation module and an adaptive Kalman filter that fuses velocity–attitude measurements. The design rationale and implementation details of each part are elaborated below.

### 3.1. Attention-LSTM Compensation Module

In practice, the master/slave lever-arm effect manifests as a mix of unknown initial offsets, piece-wise constants, abrupt jumps, and slowly drifting biases. These signatures stem from assembly tolerances, platform reconfiguration, structural flexure, thermal loading, and other time-varying factors. Analytical or state-augmentation methods struggle to track such characteristics quickly; performance lags are especially evident when maneuver excitation is weak or a lever-arm step occurs. To close this gap, we introduce a feed-forward compensator based on Attention-enhanced LSTM. Operating solely on a short sliding window of slave IMU data, the network implicitly learns the dynamic lever-arm signature in real time and outputs a corrective linear-velocity increment. The compensated velocity difference, together with the attitude difference, is then fed to an adaptive Kalman filter, boosting overall alignment accuracy.

#### 3.1.1. Training Data Generation and Disturbance Design

As the rigid–flexible lever-arm model shows, the combined action of static offset and dynamic deformation injects a deterministic yet intractable systematic error into the navigation frame velocity difference between the master and slave INS. In field applications, the time-varying equivalent lever arm cannot be measured directly, nor can the lever-arm-induced velocity error be isolated online from other sensor errors. We therefore recast the problem as a purely data-driven sequence-to-sequence mapping: during training, short windows of slave IMU data served as input while the lever-arm-induced fraction of the master/slave velocity difference acted as the supervised target. The network learned the nonlinear transformation from the IMU snippet to this velocity error; at run-time, only the same short IMU window was required to predict and cancel the error, eliminating any need for explicit lever-arm observations.

First, the network input was restricted to a short IMU snippet from the slave INS. At discrete epoch $t_{k}$, the single-step observation vector is the 9-D quantity:

(12) $$z_{k}={\left[\omega_{k}^{b},\alpha_{k}^{b},f_{k}^{b}\right]}^{T}$$

where $\omega_{k}^{b}$ is the three-axis angular rate measured by the slave INS, $\alpha_{k}^{b}$ is the angular acceleration obtained by differencing consecutive angular-rate samples, and $f_{k}^{b}$ is the three-axis specific force. Let the sliding-window length be L; then, the input tensor at epoch k is defined as

(13) $$X_{k}={\left[z_{k-T+1},\ldots ,z_{k}\right]}^{T}\in {\mathbb{R}}^{T\times 9}$$

**Figure 2:**
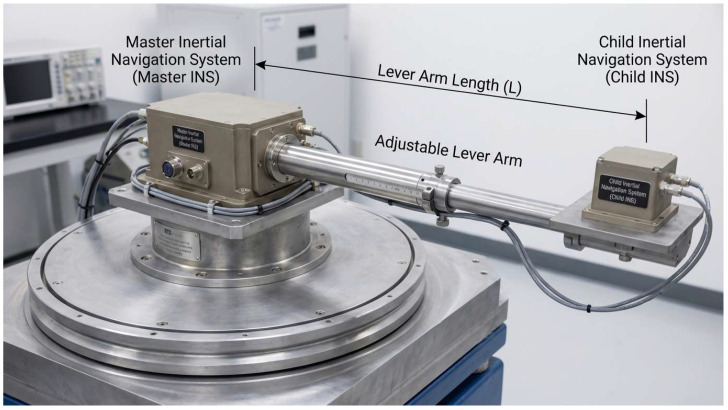
Schematic diagram of the hardware-in-the-loop experimental platform.

The label vector $Y_{k}\in {\mathbb{R}}^{3}$ denotes the systematic lever-arm contribution to the master/slave velocity difference in the navigation frame—the ground-truth velocity error that the feed-forward module must cancel. Since this error cannot be measured directly, we created training labels offline using a hybrid hardware-in-the-loop and simulation pipeline: First, raw slave-IMU data and synchronized angular-motion commands were recorded in the three-axis table shown in Figure 2. Next, the same commands were fed into our rigid–flexible simulation framework constructed using the Precise Strapdown Inertial Navigation System (PSINS) toolbox [30] (available at http://www.psins.org.cn). Leveraging this validated MATLAB 2021a-based environment for high-precision inertial analysis, and with known master/slave geometry and lever-arm parameters, we computed both the “lever-arm-included” velocities and the ideal velocities obtained by virtually collocating the slave INS with the master. The steady, deterministic difference was taken as the lever-arm-induced velocity error, time-aligned with the recorded IMU sequence, and used as the supervision signal.

It should be emphasized that the labeling procedure was performed entirely offline, relying on prior modeling of the representative platform’s geometry and flexible dynamics. Once training was complete, the network could estimate the lever-arm-induced velocity error in real time using only a short snippet of slave-IMU data; no explicit measurement or online identification of the equivalent lever arm was required during operation.

To improve training stability and remove dimensional effects, both inputs and labels were subjected to per-channel Z-score normalization:

(14) $$\begin{array}{l} {\tilde{X}}_{k}=\mathrm{Norm}(X_{k};\mu_{X},\sigma_{X})\\ {\tilde{Y}}_{k}=\mathrm{Norm}(Y_{k};\mu_{Y},\sigma_{Y}) \end{array}$$

where $\mu_{X},\sigma_{X},\mu_{Y},\sigma_{Y}$ were obtained from the training set statistics and used for de-normalization during the online phase.

Data splitting follows a “stratify-by-scenario” rule: sequences corresponding to different maneuver types or different equivalent-lever-arm configurations never overlap across training, validation, or test sets, thus preventing information leakage and over-fitting. After the above steps, we obtained the sample sets:

(15) $$D={\left\{({\tilde{X}}_{k},{\tilde{Y}}_{k})\right\}}_{k=1}^{N},$$

By minimizing the mean-square-error loss function,

(16) $$L=\frac{1}{N}\sum_{k=1}^{N}{\left\|{\hat{Y}}_{k}-{\tilde{Y}}_{k}\right\|}_{2}^{2}$$

we learn the nonlinear mapping from the short-duration slave-IMU sequence to the systematic velocity error, where ${\left\|\cdot \right\|}_{2}^{2}$ denotes the squared Euclidean norm.

#### 3.1.2. Network Architecture Design

The proposed Attention-LSTM network consists of an LSTM layer, a self-attention layer, and a fully-connected output layer. The input layer comprises slave-IMU angular velocity ω(t), angular acceleration $\dot{\omega }(t)$, and specific force f(t), giving nine channels; the target is the 3-D lever-arm velocity error $\delta v_{\mathrm{ture}}(t)$.

First, the LSTM extracts temporal features and yields the hidden-state sequence:

(17) $$H=[h_{1},h_{2},\ldots ,h_{t},\ldots ,h_{T}]$$

where $h_{t}$ denotes the hidden-state vector at the corresponding time instant, and *T* is the sliding-window length.

To adaptively emphasize key dynamic segments under non-stationary disturbances, a self-attention mechanism is introduced:

(18) $$\left\{\begin{array}{c} e_{t}=v^{T}\tanh(W_{h}h_{t}+b_{h})\\ \alpha_{t}=\frac{\exp(e_{t})}{\sum_{j=1}^{T}\exp(e_{j})} \end{array}\right.$$

Here, $W_{h}$ is the weight matrix of the attention layer; $b_{h}$ is the bias vector; v is the attention score vector; $e_{t}$ is the attention energy value at time *t*; and $\alpha_{t}$ is the corresponding attention weight at time *t*.

The final context feature representation is then generated from the attention weights:

(19) $$c=\sum_{t=1}^{T}\alpha_{t}h_{t}$$

Finally, the context feature ***c*** is passed through a fully-connected layer to yield the predicted lever-arm velocity error:

(20) $$V_{\mathrm{pred}}=W_{c}c+b_{c}$$

where $W_{h}$ is the weight matrix of the output layer, and $b_{h}$ is the output-layer bias.

After offline training, the Attention-LSTM model accepts online sliding-window snippets $X_{k}$ of the slave-IMU data and produces the normalized estimate ${\hat{\tilde{y}}}_{k}$ of the navigation frame velocity difference. Using the training-set statistics $\left(\mu_{Y},\sigma_{Y}\right)$, this estimate is de-normalized to obtain

(21) $${\hat{\Delta v}}^{\;n}(t_{k})=\sigma_{Y}⊙{\hat{\tilde{y}}}_{k}+\mu_{Y}$$

where ⊙ denotes the Hadamard product.

Treating this prediction as the velocity error introduced by the equivalent lever-arm effect, we cancel it in a feed-forward manner to obtain the online velocity observation:

(22) $$z_{v}(t_{k})=(v_{s}^{n}(t_{k})-v_{m}^{n}(t_{k}))-{\hat{\Delta v}}^{\;n}(t_{k})$$

Thereafter, $z_{v}(t_{k})$, together with the master/slave attitude difference, forms the velocity–attitude joint observation vector that is fed into the adaptive Kalman filter (AKF).

#### 3.1.3. Network Training Parameter Configuration

The detailed parameter settings used during training are listed in Table 2.

The sliding window length was empirically optimized. Preliminary experiments with varying window sizes (ranging from 10 to 50) indicated that a length of 20 provided the best trade-off between feature extraction capability and inference latency. Smaller windows failed to capture sufficient temporal dependencies of the flexible deformation, while larger windows did not yield significant accuracy improvements but increased the computational cost.

### 3.2. Filter Design

To interface with the compensation term derived in the previous section, we adopt the classic 18-state error vector to characterize the slave INS errors relative to the master INS [31,32,33]:

(23) $$x={\left[\delta \psi^{n}\;\;\delta v^{n}\;\;\delta r^{n}\;\;b_{g}\;\;b_{a}\;\;\theta_{\mathrm{ins}}\right]}^{T}\in {\mathbb{R}}^{18}$$

where $\delta \psi^{n}\;$ denotes the small-attitude error, $\delta v^{n},\delta r^{n}$ are the velocity and position errors in the navigation frame, $b_{g},b_{a}$ are the gyroscope and accelerometer biases, and $\theta_{\mathrm{ins}}$ represents the misalignment angles.

The system state equation is obtained by discretizing the dynamics of the above error states and can be written in the linear form:

(24) $$x_{k+1}=\Phi_{k}x_{k}+\Gamma_{k}w_{k}$$

Here, $\Gamma_{k}$ is the process noise matrix, specifically:

(25) $$\Gamma_{k}=\left[\begin{array}{ccccc} 0 & -[\omega_{m}^{n}\times ] & 0 & -C_{s}^{n} & 0\\ 0 & 0 & -[\omega_{m}^{n}\times ] & 0 & -C_{s}^{n}\\ 0 & I_{3} & 0 & 0 & 0\\ 0 & 0 & 0 & 0 & 0\\ 0 & 0 & 0 & 0 & 0 \end{array}\right]$$

where $\Phi_{k}$ is the state transition matrix, and $w_{k}$ is the process noise vector. This captures the uncertainty introduced at every propagation step by external disturbances or modeling errors and is usually modeled as a zero-mean Gaussian white-noise process:

(26) $$W_{k}\sim N(0,Q_{k})$$

Here, $Q_{k}$ denotes the process noise covariance matrix.

In this paper, we adopt a fully actuated state-space modeling strategy, so the process-noise vector is defined globally as

(27) $$w_{k}={\left[w_{\psi }^{T}\;w_{v}^{T}\;\;w_{r}^{T}\;\;w_{bg}^{T}\;\;w_{ba}^{T}\;w_{ins}^{T}\right]}^{T}$$

The observation equation consists of two parts: velocity matching and attitude matching.

The velocity observation is formed by the difference between the compensated master-INS velocity ${\hat{v}}_{m}^{n}(k)$ and the slave-INS velocity $v_{s}^{n}(k)$:

(28) $$z_{v}(k)=v_{s}^{n}(k)-{\hat{v}}_{m}^{n}(k)$$

The attitude measurement term employs the skew-symmetric part of the direction cosine matrix difference between the master and slave INS as the observation vector:

(29) $$z_{\phi }(k)=\frac{1}{2}\left[{\left(C_{m}^{n}(k)C_{s}^{n}{\left(k\right)}^{T}-C_{s}^{n}(k)C_{m}^{n}{\left(k\right)}^{T}\right)}^{\vee }\right]$$

where ${\left(\cdot \right)}^{\vee }$ denotes its inverse mapping. The joint observation vector is

(30) $$Z_{k}=\left[\begin{array}{c} z_{\phi }(k)\\ z_{v}(k) \end{array}\right]=H_{k}X_{k}+V_{k}$$

The basic filter recursion formulas are as follows:

Prediction phase:

(31) $$\left\{\begin{array}{l} X_{k|k-1}=F_{k-1}X_{k-1|k-1}\\ P_{k|k-1}=F_{k-1}P_{k-1|k-1}F_{k-1}^{T}+Q_{k-1} \end{array}\right.$$

Update phase:

(32) $$\left\{\begin{array}{l} K_{k}=P_{k|k-1}H_{k}^{T}{\left(H_{k}P_{k|k-1}H_{k}^{T}+R_{k}\right)}^{-1}\\ X_{k|k}=X_{k|k-1}+K_{k}(Z_{k}-H_{k}x_{k|k-1})\\ P_{k|k}=(I-K_{k}H_{k})P_{k|k-1} \end{array}\right.$$

Traditional Kalman filtering assumes that the statistical properties of process and measurement noise are known and fixed; however, during actual transfer alignment, vehicle maneuvers and environmental changes often cause these properties to vary. To improve estimation accuracy and robustness, we embed an adaptive mechanism based on the innovation sequence. The adaptive Kalman filter uses the innovation (residual) sequence to update the measurement noise covariance in real time: by setting minimum and maximum bounds $R_{\min}$ and $R_{\max}$, the current residual dynamically adjusts $R_{k}$, thereby reducing attitude error. During the update, when the residual increases, the estimated $R_{k}$ approaches $R_{\max}$, reducing the influence of the current measurement; when the residual decreases, $R_{k}$ approaches $R_{\min}$, increasing trust in the measurement. The adaptive adjustment formula for the measurement noise covariance is

(33) $$\left\{\begin{array}{l} e_{k}=Z_{k}-H_{k}X_{k|k-1}\\ {\hat{R}}_{k}=(1-\beta ){\hat{R}}_{k-1}+\beta (e_{k}e_{k}^{T}) \end{array}\right.$$

where $e_{k}$ is the observation residual at step ***k***; β∈(0,1] is the residual fusion coefficient, taken as β=0.05 in this paper to control the influence weight of the new residual on the estimate; ${\hat{R}}_{k}$ is the estimated current observation noise covariance matrix, and to prevent the estimate from becoming too large or too small, the diagonal elements of ${\hat{R}}_{k}$ are constrained between $R_{\max}$ and $R_{\min}$:

(34) $$R_{k}=\mathrm{diag}(\mathrm{clip}(\mathrm{diag}({\hat{R}}_{k}),R_{\min},R_{\max}))$$

where diag(⋅) denotes extracting the diagonal elements into a vector, and clip(⋅) denotes element-wise clamping to the given interval. Finally, $R_{k}$ replaces the observation noise covariance in Equation (32) to complete the AKF update.

In summary, the velocity error correction and adaptive filtering strategy based on Attention-LSTM compensation have been established; experiments below will verify its improvement in alignment accuracy under various maneuvers.

## 4. Experimental Analysis

To systematically evaluate the transfer alignment performance of the proposed Attention-LSTM feed-forward compensation coupled with innovation-adaptive Kalman filter in the deployment scenario of an underwater bio-inspired robot, a “hardware-in-the-loop data training and PSINS high-fidelity simulation assessment” validation framework is established. Representative slave IMU dynamic sequences are first collected on a turntable hardware-in-the-loop (HIL) platform; PSINS rigid–flexible co-simulation then generates physically meaningful lever-arm velocity error labels for these sequences to train the Attention-LSTM feed-forward compensation model. After convergence, the same PSINS environment is used to build a master and slave INS cooperative transfer alignment platform, under which the end-to-end alignment accuracy of different compensation strategies is compared across multiple typical maneuver profiles.

Specifically, the training phase adopts a hardware-in-the-loop and simulation joint labeling strategy. First, the slave IMU assembly is mounted on the turntable platform shown in Figure 2. Preset angular motion commands drive the turntable to excite the IMU’s attitude maneuvers, while raw triaxial angular rates and specific force data from the slave IMU, together with the high-precision angular motion commands, are recorded. Second, the same command set is fed into the PSINS simulation environment, where, given the known master/slave INS installation geometry and equivalent lever-arm parameters, a rigid–flexible coupling model is built to compute the master and slave navigation-frame velocities that include the equivalent lever-arm effects and the ideal reference velocities obtained by virtually translating the slave IMU to the master location. The deterministic component of their difference, arising from both the geometric lever arm and its flexible perturbation, constitutes the “lever-arm-induced velocity error” that the Attention-LSTM network must learn. Finally, the measured IMU sequence is used as the network input, and the velocity error sequence obtained from the above simulation is aligned with it by time stamp as a synchronous supervision label, thus completing supervised training without relying on online lever-arm parameter observation.

It should be noted that due to current underwater experimental constraints, complete alignment data from a real master/slave deployment environment are not yet available. Therefore, the verification phase uses a PSINS-based master/slave INS cooperative simulation platform for end-to-end evaluation. This allows systematic generation of diverse motion profiles, lever-arm configurations, and flexible disturbances under controlled conditions to test cross-scenario robustness, while the use of real turntable data during training injects authentic IMU noise and installation errors, mitigating domain shift that could arise from purely simulated training. Future work will validate the engineering applicability of the proposed method through tank tests and open-sea trials on actual underwater bio-inspired robots.

### 4.1. Transfer Alignment Simulation Environment Setup

Under the aforementioned validation framework, a representative cooperative simulation environment for the master and slave INS must first be established. The master platform represents a surface or underwater carrier equipped with a high-precision master INS, while the slave platform represents the underwater bionic robot to be deployed; before deployment, the two are connected via a hybrid rigid–flexible structure, such as a hanger, launch tube, or flexible robotic arm. Considering that the underwater bionic robot typically experiences typical maneuvers such as acceleration, turning, fin flapping, and combined maneuvers during actual missions, four representative carrier motion conditions are designed in PSINS: accelerated straight-line motion, coordinated turn, periodic fin flapping, and S-shaped maneuver. The simulation timeline adopts a unified arrangement of “uniform straight sailing for the first 5 s and the last 5 s, with the target maneuver executed in the middle segment”. The roll angle of the fin-flapping motion is generated as a sinusoidal function with given amplitude and angular frequency, and the S-shaped maneuver is constructed by alternating left–right coordinated turns. The corresponding key parameters are listed in Table 3.

**Figure 3:**
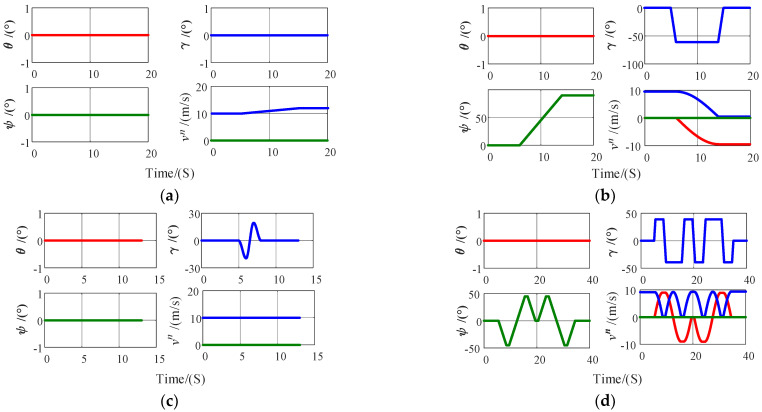
Trajectories of typical carrier motion patterns: (**a**) carrier accelerated motion; (**b**) carrier coordinated turn motion; (**c**) carrier fin-flapping motion; (**d**) carrier S-shaped motion. The red curve represents $v_{E}$, the blue curve represents $v_{N}$, and the green curve represents $v_{U}$.

The carrier trajectory is shown in Figure 3.

Here, the navigation information of the carrier’s master INS is assumed to be error-free; the simulation parameters of the slave INS on the underwater vehicle are listed in Table 4.

### 4.2. Ablation Study

**Figure 4:**
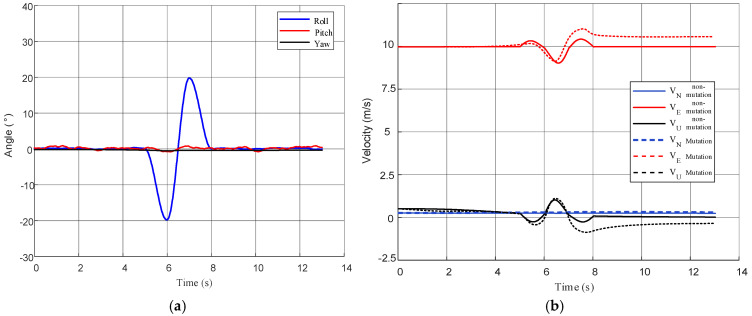
Simulation data trajectories of the slave INS: (**a**) slave INS attitude; (**b**) slave INS velocity.

Ablation analysis was conducted on the key modules of the proposed method, focusing on velocity observation construction and lever-arm compensation strategies. The simulation scenario selected is the fin-flapping motion trajectory, where the carrier executes periodic roll excitation with fixed amplitude and frequency to fully activate lever-arm-related states. Figure 4 first presents the attitude and velocity simulation results of the slave INS under this condition. It can be observed that the attitude maintains significant periodic oscillations throughout the alignment period, and the velocity also exhibits obvious non-stationary variations, providing favorable conditions for evaluating the performance of various compensation strategies under strong excitation scenarios.

**Figure 5:**
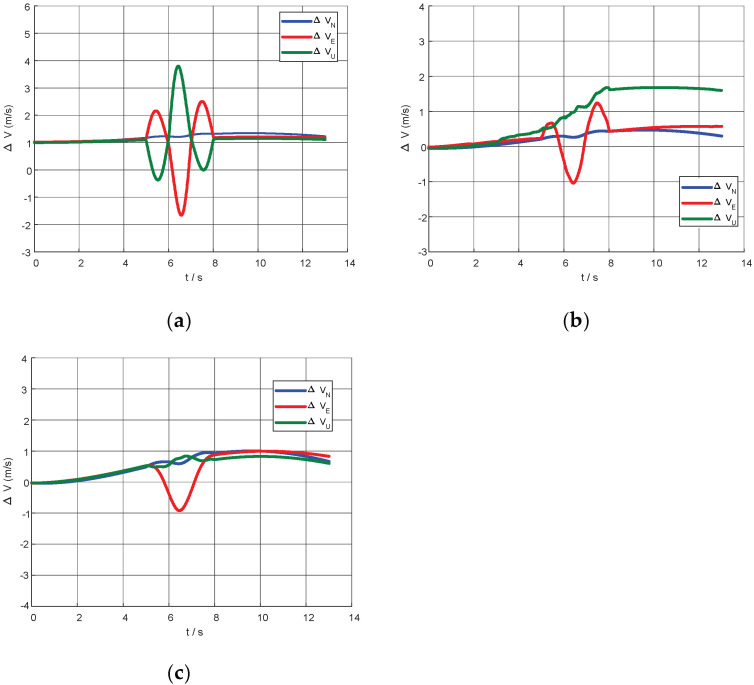
Comparison of master and slave INS velocity difference curves: (**a**) velocity error with no compensation; (**b**) velocity error with Attention-LSTM compensation; (**c**) velocity error with physical compensation.

On this basis, two typical scenarios are considered: “no lever-arm abrupt change” and “with rigid lever-arm abrupt change.” For the former, the equivalent lever-arm between the master and slave INS was assumed to remain constant throughout the entire alignment process. Under this condition, the physical model was complete and the lever-arm parameters were fully calibrated, representing the ideal application condition for traditional rigid body computational compensation methods. Figure 5 shows the time evolution curves of the master/slave INS velocity difference under three strategies: no compensation, physical lever-arm compensation, and Attention-LSTM compensation. Without compensation, the master/slave velocity difference exhibited large-amplitude periodic fluctuations during the entire alignment period, indicating that lever-arm effects and other common-mode errors dominated the velocity observations. After applying physical compensation using rigid lever-arm parameters, the velocity difference curve became significantly smoother, with greatly reduced amplitude; the residual error was mainly high-frequency noise. The Attention-LSTM compensation, without explicitly inputting lever-arm parameters, produced a velocity difference curve whose overall shape is highly consistent with physical compensation, with similar fluctuation amplitude. This demonstrates that the network successfully learned the main dynamic characteristics of lever-arm-induced velocity errors from the inertial measurement sequences and can approach the optimal compensation effect of the analytical model under the “no abrupt change” scenario.

**Figure 6:**
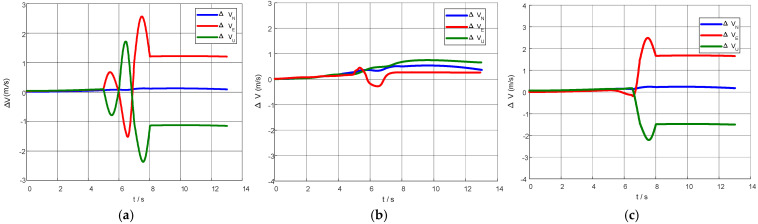
Velocity error curves before and after the abrupt lever-arm change: (**a**) velocity error with no compensation; (**b**) velocity error with Attention-LSTM compensation; (**c**) velocity error with physical compensation.

To examine the algorithm’s adaptability to lever-arm abrupt change, a step-type rigid lever-arm abrupt change in the same direction was imposed on the above fin-flapping trajectory: the equivalent lever-arm length jumped from A = 1 to A = 2 (simulating a sudden mechanical extension or reconfiguration of the deployment boom, where the lever-arm magnitude doubles while the direction remains unchanged). The conventional physical compensation strategy kept using the initially calibrated value throughout the whole alignment and performed no on-line correction for the abrupt change, so significant model mismatch appeared after the jump. Figure 6 plots the master/slave INS velocity differences versus time for the three compensation schemes. Without compensation, the difference still exhibited large periodic oscillations; physical compensation showed evident mismatch: the bias of the velocity difference rose sharply after the jump and stayed at a high level for a long time, yielding large compensation error. By contrast, the Attention-LSTM compensation underwent only a brief transient after the jump and soon returned to a low-amplitude oscillation band, leaving a residual bias clearly smaller than that of the physical scheme.

This indicates that once analytical compensation based on fixed lever-arm parameters encounters changes beyond the calibration range, such as structural reconfiguration, mounting position adjustment, or flexible deformation, its model fails globally and cannot be quickly corrected by filter feedback. Attention-LSTM, however, extracts features from short IMU time slices and estimates and cancels systematic velocity terms in a feed-forward manner. Even when the external equivalent lever-arm undergoes a step change, the network can still adapt on the basis of the learned dynamic patterns, demonstrating stronger fault tolerance and generalization under abrupt change conditions.

The results show that, without accounting for the lever-arm effect, the master/slave velocity difference persisted as large periodic oscillations. Under abrupt lever-arm change, physical compensation degraded markedly—once the model fails, the computed correction itself prevents convergence of the velocity difference. In contrast, the Attention-LSTM model maintained satisfactory compensation performance, demonstrating superior fault tolerance and generalization.

### 4.3. Transfer Alignment Experiments with Different Compensation Strategies

After completing the local ablation analysis, this paper further examines the comprehensive effects of different velocity compensation strategies within the full “velocity–attitude joint matching and adaptive Kalman filter” transfer alignment chain. Specifically, the three schemes of “no compensation,” “physics-based compensation (Physics + AKF),” and “Attention-LSTM compensation (Attention-LSTM + AKF)” were separately integrated into the same AKF, and their estimation performance for the triaxial misalignment angles and the mounting-error angles under several typical maneuvering trajectories was compared.

**Figure 7:**
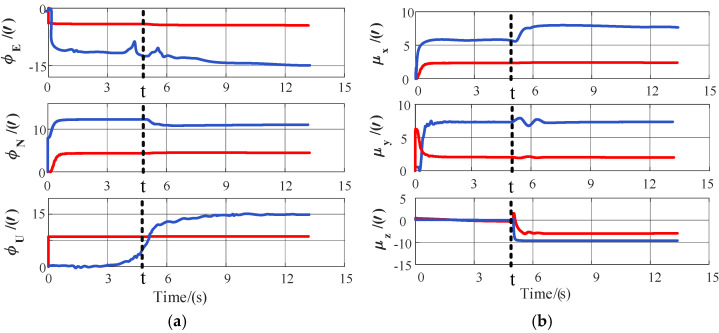
Estimation accuracy of the velocity–attitude joint transfer alignment: (**a**) misalignment angle error; (**b**) mounting angle error. The red curve represents Attention-LSTM compensation, and the blue curve represents physics-based compensation.

First, the wing-rock maneuver was selected to analyze how a sudden lever-arm change affects downstream attitude estimation. Figure 7 shows the time histories of the Strapdown Inertial Navigation System (SINS) misalignment angle errors and mounting angle errors for this scenario; a rigid lever-arm step was injected at mid-run. It can be seen that with Physics + AKF, the velocity observation retained a step-induced bias that the filter could not eliminate quickly, so all three misalignment angles and the mounting angles exhibited pronounced instantaneous peaks, maintained noticeable residual offsets for a long period, displayed heavy convergence tails, and suffered from markedly larger steady-state errors. After switching to Attention-LSTM + AKF, the feed-forward compensation suppressed the velocity bias at the observation level. The misalignment and mounting angle curves exhibited only limited fluctuations around the step instant, dropped back promptly, and settled into a small-error band. Overall convergence time was noticeably shortened, while overshoot and steady-state residuals were both greatly reduced.

To comprehensively evaluate the robustness of the proposed method under different maneuvering conditions, Table 5 summarizes the alignment results of the two compensation schemes under four typical trajectories: straight-line acceleration, coordinated turn, wing rock, and S-curve maneuvers. The table presents the estimation errors for misalignment angles and mounting angles in each channel.

Table 5 shows that, when a sudden lever-arm length change occurred, Physics + AKF yielded poor overall accuracy for both horizontal misalignment and mounting angles. In some maneuvers, the error components even exhibited noticeable biases, which is detrimental to subsequent navigation. By contrast, Attention-LSTM + AKF consistently decreased the error amplitude in every channel under all four maneuvering conditions, and the reduction was especially pronounced during the combined turning and S-curve flight.

To facilitate a quantitative comparison of the alignment accuracy achieved by different methods, we introduced the tri-axial composite root-mean-square (RMS) error as the metric. For each maneuver, the composite RMS values corresponding to the misalignment angle error and mounting angle error are defined as follows:

(35) $$\left\{\begin{array}{l} E_{\phi }^{\left(m,j\right)}=\sqrt{\frac{{\left(\delta \phi_{E}^{\left(m,j\right)}\right)}^{2}+{\left(\delta \phi_{N}^{\left(m,j\right)}\right)}^{2}+{\left(\delta \phi_{U}^{\left(m,j\right)}\right)}^{2}}{3}}\\ E_{\mu }^{\left(m,j\right)}=\sqrt{\frac{{\left(\delta \mu_{x}^{\left(m,j\right)}\right)}^{2}+{\left(\delta \mu_{y}^{\left(m,j\right)}\right)}^{2}+{\left(\delta \mu_{z}^{\left(m,j\right)}\right)}^{2}}{3}} \end{array}\right.$$

Taking Physics + AKF as the benchmark, the relative reduction for the ***j***-th maneuver is defined as follows:

(36) $$\left\{\begin{array}{l} \eta_{\phi }^{\left(j\right)}=\frac{E_{\phi }^{\left(\mathrm{Phys},j\right)}-E_{\phi }^{\left(\mathrm{Attn},j\right)}}{E_{\phi }^{\left(\mathrm{Phys},j\right)}}\times 100\%\\ \eta_{\mu }^{\left(j\right)}=\frac{E_{\mu }^{\left(\mathrm{Phys},j\right)}-E_{\mu }^{\left(\mathrm{Attn},j\right)}}{E_{\mu }^{\left(\mathrm{Phys},j\right)}}\times 100\% \end{array}\right.,$$

Averaging over the four maneuvering cases gives the overall mean reduction.

As shown in Table 5 and calculated by the above metrics, under the four maneuvers—acceleration, turning, fin-flapping, and S-curve—the Attention-LSTM + AKF scheme markedly reduced transfer alignment errors in all channels. The reductions in tri-axial composite misalignment angle error were about 64.1%, 64.5%, 64.6% and 64.5%, respectively; those for tri-axial composite mounting angle error were about 65.2%, 66.7%, 65.3% and 66.5%. Averaged across all runs, the composite RMS misalignment angle of Physics + AKF was roughly 14.5′, whereas the proposed method yielded only 5.2′—an average reduction of ~64%. The composite RMS mounting angle error dropped from 8.8′ to 3.0′, an average reduction of ~66%. Therefore, in the presence of unknown or abruptly changing lever arms, the joint alignment scheme based on Attention-LSTM feed-forward compensation plus AKF significantly improves both convergence speed and steady-state accuracy of transfer alignment.

## 5. Discussion and Outlook

Under the velocity–attitude joint matching framework, this study compares the conventional Physics + AKF with the proposed Attention-LSTM + AKF. Simulations incorporating flexible lever-arm disturbances and installation errors demonstrated that the proposed method consistently reduced the composite RMS misalignment and mounting angle errors by approximately 64% and 66%, respectively. This indicates that the data-driven strategy significantly outperforms the physical model in handling complex bio-inspired maneuvers.

Regarding the applicability in engineering practice, several critical design choices and constraints are addressed. First, the Extended-State Kalman Filter (ESKF) was not selected as a baseline because it relies on linearization and struggles to track the high-frequency, non-linear oscillations (0.5–2 Hz) inherent to bio-inspired fin flapping. In contrast, the proposed LSTM directly maps these non-linear kinematic features without the lag associated with state augmentation. Second, concerning the simulation-to-reality gap, while HIL setups cannot perfectly replicate open-water hydrodynamics, the lever-arm effect is fundamentally a kinematic phenomenon, ensuring the learned mapping remains valid provided sensor characteristics are consistent. Third, regarding signal processing, we leveraged the LSTM’s gating mechanisms as an implicit filter to suppress high-frequency measurement noise amplified by differentiation, thereby avoiding the phase lag of explicit low-pass filters. Finally, the proposed lightweight architecture ensures real-time feasibility, allowing millisecond-level inference on standard embedded processors (e.g., STM32 or Jetson Nano) to satisfy 50–100 Hz navigation loops.

Future work will focus on enhancing the model’s generalizability and robustness. Directions include (1) introducing physics-informed priors to improve interpretability; (2) integrating external cues such as DVL or visual odometry for long-term navigation; and (3) systematically investigating the algorithm’s robustness under conditions of sensor degradation and varying water current disturbances.

## Figures and Tables

**Table 1:** Definition of basic symbols used in this paper.

Symbol	Meaning
i	Inertial frame
b	Body frame
n	Navigation frame
$r_{m}^{i}$	Position vector of the master inertial navigation system (INS) in the inertial frame
$r_{s}^{i}$	Position vector of the slave INS in the inertial frame
$r_{sm}^{b}$	Displacement vector of the slave INS relative to the master INS, expressed in the body frame
$\omega_{ib}^{b}$	Angular velocity of the body relative to the inertial frame, represented in the body frame
$C_{b}^{i}$	Direction cosine matrix from the body frame to the inertial frame
$v_{m}^{n}$ $v_{s}^{n}$	Velocities of the master and slave INS in the navigation frame
$a_{m}^{n}$ $a_{s}^{n}$	Accelerations of the master and slave INS in the navigation frame

**Table 2:** Configuration of network training parameters.

Parameter	Setting
Batch size	16
Epoch	20
Learning rate	0.001
Number of LSTM hidden layers	2
Hidden layer neurons	64
Optimizer	AdamW
Hidden dimension	128
Attention dimension	64
Dropout	0.1
Window length	20
Training/validation/test	70%/15%/15%

**Table 3:** Settings for carrier maneuver simulation codes.

Carrier Maneuver	Simulation Parameter
Acceleration	5 s~15 s: 2 m/s^2^ forward acceleration
Coordinated turn	5 s~15 s: 90° left turn
Fin flapping	5 s~8 s: amplitude 20°, frequency 0.5 Hz
S-curve	5 s~35 s: S-shaped maneuver

**Table 4:** Parameter settings for the simulation experiment.

Parameter Term	Content	Parameter Setting
Slave INS Gyroscope	Gyro Drift $\varepsilon^{s}$	0.02°/s
	Angle Random Walk $\varepsilon_{w}^{s}$	$0.15{}^{\circ}/\sqrt{h}$
Slave INS Accelerometer	Accelerometer Bias $\nabla^{s}$	500 μg
	Velocity Random Walk $\nabla_{w}^{s}$	1000 μg
Initial Bias	Attitude Error Δf	0.4°, 0.4°, −0.8°
Body Frame Error	Installation Error Angle μ	0.5°, 0.5°, −0.5°
Flexural Deformation	Process Standard Deviation σ	6′, −10′, 7′
	Correlation Time τ	0.5 s, 0.4 s, 10 s
Master INS Initial Conditions	Attitude θ,γ,ψ Velocity $v_{E},v_{N},v_{U}$	0°, 0°, 0° 0 m/s, 10 m/s, 0 m/s
Initial Lever Arm	$r_{sm}^{b}$	[1.1, 0.8, 0.2]^*T*^ m

**Table 5:** Comparison of transfer alignment estimation accuracy on the PSINS simulation platform.

	Physics + AKF		Attention-LSTM + AKF	
Trajectory	Misalignment angle error $\delta \phi_{E}$, $\delta \phi_{N}$, $\delta \phi_{U}$	Mounting angle error $\delta \mu_{x}$, $\delta \mu_{y}$, $\delta \mu_{z}$	Misalignment angle error $\delta \phi_{E}$, $\delta \phi_{N}$, $\delta \phi_{U}$	Mounting angle error $\delta \mu_{x}$, $\delta \mu_{y}$, $\delta \mu_{z}$
Acceleration	−12.0′	−8.0′	−4.2′	−2.8′
	−10.5′	7.2′	−3.8′	2.5′
	14.0′	9.5′	−5.1′	3.3′
Turning	−14.3′	−8.6′	−5.1′	2.9′
	−12.7′	−7.9′	4.6′	2.6′
	−18.5′	−10.2′	−6.5′	3.4′
Fin-Flapping	−13.6′	8.4′	−4.7′	2.9′
	11.4′	7.4′	4.3′	2.6′
	17.2′	−9.8′	6.0′	−3.4′
S-curve	−15.1′	8.9′	5.3′	3.0′
	−13.8′	8.1′	−4.9′	2.7′
	−19.0’	−10.5’	−6.8’	3.5’

## Data Availability

The original contributions presented in this study are included in the article material. Further inquiries can be directed to the corresponding author.
